# Accuracy of Critical Care Ultrasonography Plus Arterial Blood Gas Analysis Based Algorithm in Diagnosing Aetiology of Acute Respiratory Failure

**DOI:** 10.2478/jccm-2023-0006

**Published:** 2023-02-08

**Authors:** Rajesh Panda, Saurabh Saigal, Rajnish Joshi, Abhijit Pakhare, Ankur Joshi, Jai Prakash Sharma, Sahil Tandon

**Affiliations:** 1 All India Institute of Medical Science - Bhopal, Madhya Pradesh, India

**Keywords:** critical care ultrasound, arterial blood gas, chest X ray, correlation, dyspnoea, ICU, lung ultrasound, acute respiratory failure, algorithm, critically ill

## Abstract

**Introduction:**

Lung ultrasound when used in isolation, usually misses out metabolic causes of dyspnoea and differentiating acute exacerbation of COPD from pneumonia and pulmonary embolism is difficult, hence we thought of combining critical care ultrasonography (CCUS) with arterial blood gas analysis (ABG).

**Aim of the study:**

The objective of this study was to estimate accuracy of Critical Care Ultrasonography (CCUS) plus Arterial blood gas (ABG) based algorithm in diagnosing aetiology of dyspnoea. Accuracy of traditional Chest X-ray (CxR) based algorithm was also validated in the following setting.

**Methods:**

It was a facility based comparative study, where 174 dyspneic patients were subjected to CCUS plus ABG and CxR based algorithms on admission to ICU. The patients were classified into one of five pathophysiological diagnosis 1) Alveolar( Lung-pneumonia)disorder ; 2) Alveolar (Cardiac-pulmonary edema) disorder; 3) Ventilation with Alveolar defect (COPD) disorder ;4) Perfusion disorder; and 5) Metabolic disorder. We calculated diagnostic test properties of CCUS plus ABG and CXR based algorithm in relation to composite diagnosis and correlated these algorithms for each of the defined pathophysiological diagnosis.

**Results:**

The sensitivity of CCUS and ABG based algorithm was 0.85 (95% CI-75.03-92.03) for alveolar (lung) ; 0.94 (95% CI-85.15-98.13) for alveolar (cardiac); 0.83 (95% CI-60.78-94.16) for ventilation with alveolar defect; 0.66 (95% CI-30-90.32) for perfusion defect; 0.63 (95% CI-45.25-77.07) for metabolic disorders.Cohn’s kappa correlation coefficient of CCUS plus ABG based algorithm in relation to composite diagnosis was 0.7 for alveolar (lung), 0.85 for alveolar (cardiac), 0.78 for ventilation with alveolar defect, 0.79 for perfusion defect and 0.69 for metabolic disorders.

**Conclusion:**

CCUS plus ABG algorithm is highly sensitive and it’s agreement with composite diagnosis is far superior. It is a first of it’s kind study, where authors have attempted combining two point of care tests and creating an algorithmic approach for timely diagnosis and intervention.

## Introduction

Dyspnoea is one of the commonest symptom in critically ill patients admitted to Intensive care unit (ICU). Dyspnoea as commonly believed is not only limited to alveolar pathologies (pneumonias) but is a common symptom in cardiac disorders, acute exacerbation of chronic obstructive pulmonary disease (COPD), pulmonary embolism and few metabolic disorders [1]. In recent years, the expansion of domain of lung ultrasound (LUS) has contributed extensively in the diagnosis of acute dyspnoea [2]. Combining LUS with Cardiac Ultrasound (CUS), inferior vena cava (IVC) assessment and deep vein thrombosis (DVT) scan gives intensivist a comprehensive picture which in turn helps in early decision making [3]. According to current literature, the LUS was found to be superior to Chest X ray in patients with pneumonia and cardiogenic pulmonary oedema [4]. In COPD patients, till date only four studies have evaluated the efficacy of LUS which was inferior to its efficacy in pneumonia and pulmonary oedema [4]. Lung ultrasound usually misses out metabolic causes of dyspnoea and differentiating acute exacerbation of COPD from pneumonia and pulmonary embolism is also tricky, hence we thought of combining Critical Care Ultrasonography (CCUS) with Arterial blood gas analysis (ABG). ABG analysis doesn’t help in classifying dyspnoea directly, the carbon dioxide levels, oxygenation and metabolic parameters (pH and HCO3) provide valuable inputs into pathophysiology of the disease [5,6]. It is a first of it’s kind study, where authors have attempted combining two point of care tests i.e. bed side ultrasound and arterial blood gas analysis for diagnosing aetiology of dyspnoea and creating an algorithmic approach for timely diagnosis and intervention. We undertook this study to assess accuracy of Critical Care Ultrasonography (CCUS) plus ABG based algorithm in diagnosing aetiology of dyspnoea.

## Materials and methods

Design and Ethical statement: This is a facility based comparative study, which was conducted in a fifteen bedded Emergency Medical ICU of a tertiary care teaching hospital of Central India. The unit is equipped with portable M-turbo (Sonosite, 30^th^ Drive, SE, Bothell, USA) ultrasound machine and blood gas analyser machine (Roche, South San Francisco, California, USA). As per the ICU protocol, post admission vitals (heart rate, respiratory rate [RR], non-invasive blood pressure, ECG, oxygen saturation) were immediately assessed. A peripheral line was secured, an arterial blood gas sample was withdrawn and Complete Blood Count, Renal function test with electrolytes and liver function tests were sent along with chest x-ray. On admission, CCUS scan was performed by one of the three trained intensivists available in the unit. All investigation findings, treatment decisions, and composite diagnosis were recorded on the ICU treatment charts. The study design( with a request for waiver of consent) was approved by Institutional Ethical Committee of AIIMS Bhopal with LOP no IHECPGRDM054.

### Inclusion criteria

We included all patients aged more than 18 years presenting with dyspnoea (defined as breathlessness or laboured breathing or difficulty in breathing). The duration of ICU stay of 24 h or longer was required for inclusion, assuming that complete diagnostic information in form of blood investigations, Chest X ray, CCUS would be available till that time. We excluded pregnant patients, patients with incomplete data (CCUS/CxR) and uninterpretable CxR or CCUS scan.

### Study procedures

**Fig. 1:**
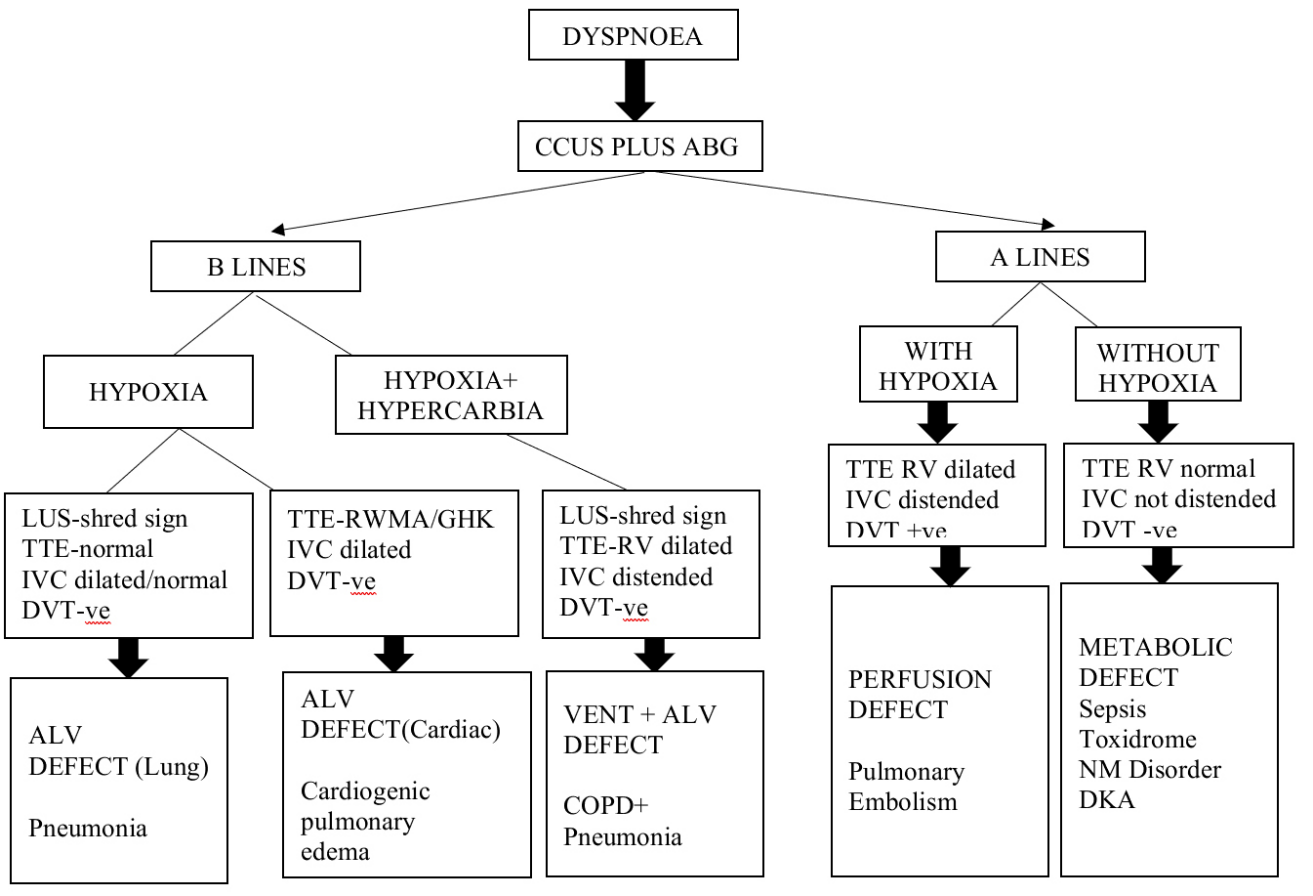
Critical Care Ultrasonography plus arterial blood gas analysis based algorithm

**Fig. 2:**
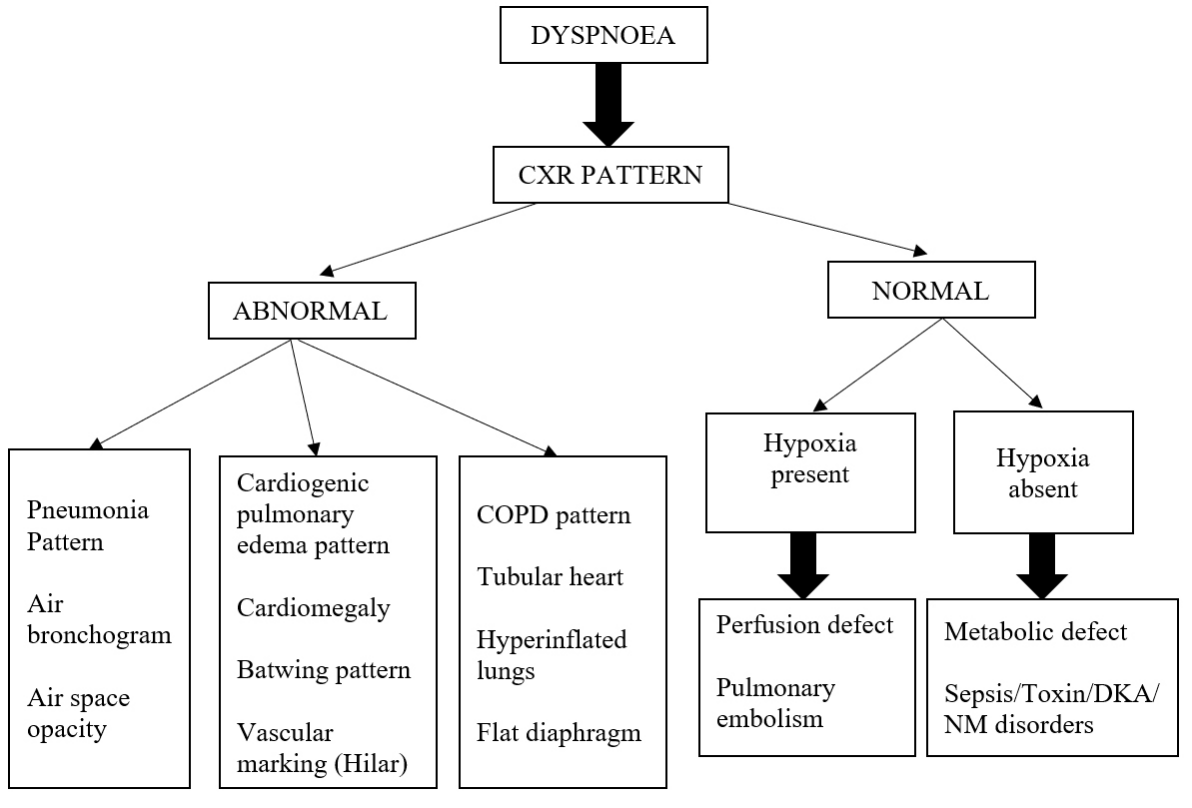
Chest X ray based algorithm

The patients were subjected to CCUS plus ABG based algorithm in which we collected information about LUS patterns , TTE (Trans-thoracic Echocardiography), IVC along with ABG on admission to ICU. On the basis of the findings as described in Figure 1 the patients were classified into one of the described pathophysiological domains. The bedside CCUS plus ABG based diagnosis was made by one of the three intensivists, who was present at the time of admission (all with equipoise training in CCUS) . The diagnosis was noted on data collection sheet and then put in a sealed envelope. Once the Chest X-ray was done, the image was sent to two independent physicians not directly related to patient care , who made the diagnosis on the basis of Chest X ray based algorithm as described in Figure 2 and diagnosis was noted on data collection sheet and then put in a sealed envelope. The team analysing the two algorithms were different. The composite diagnosis is the final diagnosis made by two critical care consultants at the end of 48 hours after carefully interpreting clinical and investigational data which included CT scan, Echocardiography and blood investigations. Once the study was completed , the sealed envelopes were opened and correlation of the CCUS plus ABG based algorithm vs Composite diagnosis, CxR algorithm vs Composite diagnosis and CCUS vs CxR was done for each of the pathophysiologic condition. We collected information about patient demographics (age, gender), primary admission source, severity of illness (SOFA), need for intubation, form of mechanical ventilation, vasopressor need , ICU outcome and length of ICU stay. We calculated the diagnostic test properties of each of these algorithms with composite diagnosis, percent agreement and percent agreement beyond chance for each of these algorithms and final correlation of these algorithms for each of the five defined pathophysiological diagnosis (Figure 1).

### Study definitions

**Pneumonia:** Four sonographic patterns were assessed to diagnose pneumonia as defined by BLUE protocol [7]: a. C-profile – shred sign; b. Focal interstitial syndrome; c. B0-profile; d. A-profile with Posterolateral Alveolar and/or Pleural Syndrome (PLAPS).

**Acute Exacerbation of COPD:** The presence of C-profile or localized B-lines with dilated RA/RV (Right atrial/Right ventricle) with distended IVC with normal LV contractility.

**Acute heart failure:** The predominance of diffuse B-lines with LV dysfunction.

**Hypoxemia** is defined as PaO2<60mm Hg on room air, **Hypercarbia** is defined as PaCO2 >45 mmHg.

Thus on the basis of CCUS plus ABG based algorithm we created five pathophysiological categories (Figure 1). These categories are

**Alveolar defect ‐ Lung** (B-lines with shred sign, TTE- Normal, IVC- collapse/distended, Hypoxemia on ABG);

**Alveolar defect ‐ Cardiac** (Bilateral B-lines, TTE-LV dysfunction, IVC distended, Hypoxemia on ABG).

**Ventilation and alveolar defect** - Acute exacerbation of COPD (focal B lines or C-profile, TTE- RV dilated, IVC distended and Hypoxemia and Hypercarbia on ABG)

**Perfusion defect** (A- lines, with DVT scan positive and RV dilatation present on Echo and Hypoxemia on ABG)

**Metabolic defect** (A-lines, TTE- Normal, DVT scan negative and no Hypoxemia on ABG).

Similarly on the basis of X-ray based algorithm patients were classified into one of the five categories (Figure 2): i. Alveolar defect ‐ (Lung) (CxR- Air bronchogram or Air opacity with hypoxia on pulse oximetry); ii. Alveolar defect‐(Cardiac) (Bat wing appearance, cardiomegaly, hilar prominence, hypoxia on pulse oximetry); iii. Ventilation and alveolar defect (COPD) (Tubular heart, hyperinflated lungs, flattened diaphragm, and hypoxia on pulse oximetry); iv. Perfusion defect (Normal Chest X ray with hypoxia on pulse oximetry); v. Metabolic defect (Normal X ray without hypoxia on pulse oximetry).

### Statistical analysis

We estimated sample size for testing agreement between diagnosis by CCUS based algorithm and Chest Xray based algorithm with final diagnosis for pneumonia, heart failure, COPD with pneumonia separately. We used IRR package to estimate sample size. Assumptions for calculation were based on previous studies cited in review of literature. For different diagnosis sample size requirement with assumption of 80%power and 95% confidence and difference from null hypothesis agreement of 0.5; range from 50-60.Therefore for 3 major diagnosis our final total sample size was 170. Formula used was


$$\begin{array}{l} N={\left[\frac{A_{\alpha }\sqrt{Q_{01}+Q_{02}}+Z_{\beta }\sqrt{Q_{A1}+Q_{A2}}}{K_{1}-K_{2}}\right]}^{2} \end{array}$$

K_1_ and K_2_ were estimated from independent samples, each of size N. Let Q_01_ and Q_02_ be the values of Q expected under the null hypothesis and Q_A1_ and Q_A2_ be the values of Q expected under an alternative [8].

We performed a descriptive statistical analysis of demographic characteristics, diagnostic categories, and severity characteristics. We expressed measures of central tendency and dispersion for categorical variables as frequency and percentages and for continuous variables as means and standard deviation. We calculated Cohen’s Kappa for each algorithm. We estimated diagnostic test properties i.e. Sensitivity, Specificity, Positive Predictive Value, Negative Predictive Value, Likelihood ratios etc, percent agreement and percent agreement beyond chance for each algorithm. Mc Nemar’s test was used to detect difference in diagnosis with CCUS scan algorithm and X-ray based algorithm. All statistical analysis was performed using IBM SPSS Statistics for Windows, Version 21.0 (Armonk, NY: IBM Corp.) and R software IRR package.

## Results

It was a facility based comparative study, where 174 dyspneic patients were subjected to CCUS plus ABG and CxR based algorithms on admission in time period from January 2020 to September 2021. The median age was 53 years (37-64) with 61.5% males (Table 1) . 90% of our cases were from emergency department and medicine ward. Majority i.e. 94% of the cases needed invasive mechanical ventilation or NIV support. Out of 174 patients, a total of 89 patients needed invasive ventilation from which 33 (37%) belonged to alveolar defect (Lung), 23 (26%) belonged to alveolar defect (cardiac), 11(12%) belonged to COPD plus pneumonia category, 21 (23%) belonged to metabolic defect category and only 1 patient from perfusion defect category.

**Table 1:** Distribution of participants by their demographic and other characteristics

**Characteristic**	**N = 174**
Age (mean+SD, years)	53.0 (37.0-64.0)
Gender n (%)	
F -n (%)	67 (38.5%)
M -n (%)	107 (61.5%)
Source n(%)	
CCU (Coronary care unit)	4 (2.3%)
CTVS surgery) (Cardiothoracic ward vascular	2 (1.1%)
ED (Emergency department)	105 (60.3%)
ENT (Ear nose throat) ward	1 (0.6%)
EW (Eye ward)	1 (0.6%)
MW (Medicine ward)	51 (29.3%)
PW (Pulmonary ward)	10 (5.7%)
Mode of ventilation n (%)	
Facemask/NRBM/HFNO	11 (6.3%)
NIV	74 (42.5%)
INVASIVE	89 (51.1%)
SOFA on DOA to ICU Median (IQR)	6.0 (4.0-9.8)
CCUS based DX n (%)	
Alveolar (lung) defect	69 (39.7%)
Alveolar (cardiac) defect	60 (34.5%)
Ventilation plus alveolar defect	19 (10.9%)
Perfusion defect	4 (2.3%)
Metabolic defect	22 (12.6%)
CXR based DX n (%)	
Alveolar (lung) defect	71 (40.8%)
Alveolar(cardiac) defect	69 (39.7%)
Ventilation plus alveolar defect	5 (2.9%)
Perfusion defect	24 (13.8%)
Metabolic defect	5 (2.9%)
Need of vasopressors on day 1 n (%)	137 (78.7%)
LOS in ICU days Median (IQR)	6.0 (4.0, 11.0)
Composite diagnosis n (%)	
Alveolar (lung) defect	63 (36.2%)
Alveolar(cardiac) defect	55 (31.6%)
Ventilation plus alveolar defect	18 (10.3%)
Perfusion defect	6 (3.4%)
Metabolic defect	32 (18.4%)
Outcome n (%)	
Discharged	115 (66.1%)
Death	59 (33.9%)

The median SOFA score on day of admission to ICU was 6 (4.0, 9.8). 137 patients(78.7%) needed vasopressors on time of admission to ICU. The average length of stay in ICU was 6 days(4-11).The results are explained in five heads on the basis of five pathophysiological diagnosis as described above namely 1. Alveolar (Lung); 2. Alveolar (Cardiac); 3.Ventilation plus alveolar; 4. Perfusion; 5. Metabolic disorders. We demonstrated the diagnostic test properties of CCUS plus ABG based algorithm vs Composite diagnosis , CxR based algorithm vs composite diagnosis and degree of agreement between these two algorithms for each of the five pathophysiological diagnosis. (Supplementary appendix).

In all the five categories the sensitivity of CCUS plus ABG based algorithm was better than Chest X-ray based algorithm when compared with final composite diagnosis (Table 2 and Table 3). It was 85.7% vs 84.13% Alveolar (lung pathology), 94.55% vs 90.91% (Cardiogenic pulmonary edema), 83.33% vs 22.22% (Ventilation with Alveolar disorder), 66% vs 50% (Perfusion disorder), 62.65% vs 15.63% (Metabolic disorder). In cases of pneumonia and pulmonary edema the sensitivity was almost similar i.e. ability to pick up cases i.e. true positives whereas in patients on ventilation with alveolar defect i.e. Acute exacerbation of COPD the sensitivity of CCUS based USG was quite superior to CXR based algorithm. The sensitivity of CCUS based algorithm was around 60% in patients with perfusion defect and metabolic disorders which was quite high as compared to Chest X ray based algorithms (Table 4).

**Table 2:** Agreement of CCUS and CXR with Composite Diagnosis

**Characteristic**	**Alveolar (lung) defect N = 63**	**Alveolar (cardiac) defect N = 55**	**Ventilation plus alveolar defect N = 18**	**Perfusion defect N = 6**	**Metabolic defect N = 32**
**CCUS based DX**					
Alveolar (lung) defect	54 (85.7%)	3 (5.5%)	3 (16.7%)	0 (0.0%)	9 (28.1%)
Alveolar(cardiac) defect	5 (7.9%)	52 (94.5%)	0 (0.0%)	0 (0.0%)	3 (9.4%)
Ventilation plus alveolar defect	2 (3.2%)	0 (0.0%)	15 (83.3%)	2 (33.3%)	0 (0.0%)
Perfusion defect	0 (0.0%)	0 (0.0%)	0 (0.0%)	4 (66.7%)	0 (0.0%)
Metabolic defect	2 (3.2%)	0 (0.0%)	0 (0.0%)	0 (0.0%)	20 (62.5%)
**CXR based DX**					
Alveolar (lung) defect	53 (84.1%)	5 (9.1%)	10 (55.6%)	0 (0.0%)	3 (9.4%)
Alveolar(cardiac) defect	8 (12.7%)	50 (90.9%)	3 (16.7%)	3 (50.0%)	5 (15.6%)
Ventilation plus alveolar defect	1 (1.6%)	0 (0.0%)	4 (22.2%)	0 (0.0%)	0 (0.0%)
Perfusion defect	1 (1.6%)	0 (0.0%)	1 (5.6%)	3 (50.0%)	19 (59.4%)
Metabolic defect	0 (0.0%)	0 (0.0%)	0 (0.0%)	0 (0.0%)	5 (15.6%)

**Table 3:** Agreement of CCUS with CXR

**Characteristic**	**Alveolar (lung) defect N = 69**	**Alveolar (cardiac) defect N = 60**	**Ventilation plus alveolar defect N = 19**	**Perfusion defect N = 4**	**Metabolic defect N = 22**
**CXR based DX**					
Alveolar (lung) defect	53 (76.8%)	8 (13.3%)	8 (42.1%)	0 (0.0%)	2 (9.1%)
Alveolar(cardiac) defect	12 (17.4%)	50 (83.3%)	5 (26.3%)	2 (50.0%)	0 (0.0%)
Ventilation plus alveolar defect	1 (1.4%)	0 (0.0%)	4 (21.1%)	0 (0.0%)	0 (0.0%)
Perfusion defect	2 (2.9%)	2 (3.3%)	2 (10.5%)	2 (50.0%)	16 (72.7%)
Metabolic defect	1 (1.4%)	0 (0.0%)	0 (0.0%)	0 (0.0%)	4 (18.2%)

**Table 4:** Correlation of CCUS based algorithm/ Chest X ray based algorithm vs Composite diagnosis

		**Sensitivity**	**Specificity**	**PPV**	**Cohn’s Kappa**
1	CCUS vs Composite diagnosis (Alveolar- Pneumonia)	85.71% (75.03-92.3)	86.49% (78.9-91.64)	78.26 (67.18-86.36)	0.7074 (0.5593-0.8566)
2	CxR vs Composite diagnosis (Alveolar- Pneumonia)	84.13% (73.91-91.14)	83.78% (75.82-89.94)	74.65 (63.45-83.30)	0.661 (0.5131-0.8088)
3	CCUS vs CxR (Alveolar- Pneumonia)				0.59 (0.44-0.74)
4	CCUS vs Composite diagnosis (Alveolar- Cardiogenic Pulmonary Edema)	94.55% (85.15-98.13)	93.28% (87.28-98.55)	86.67 (75.83-93.08)	0.8573 (0.709-1.006)
5	CxR vs Composite diagnosis (Alveolar- Cardiogenic Pulmonary Edema)	90.91% (80.42-96.05)	84.03% (76.4-89.53)	72.46 (60.95-81.61)	0.7014 (0.551-0.8477)
6	CCUS vs CxR (Alveolar- Cardiogenic Pulmonary Edema)				0.6438 (0.49-0.75)
7	CCUS (Ventilation vs Composite with Alveolar diagnosis defect)	(60.7883.33-94.16% )	(93.5997.44-% 99)	(56.6778.95 -91.49)	(0.13980.7883 -0.9368)
8	CxR vs Composite diagnosis (Ventilation with Alveolar defect)	22.22% (9.001-45.22)	99.36% (96.46-99.89)	80 (37.55-96.38)	0.3171 (0.1973-0.4369)
9	CCUS vs CxR (Ventilation with Alveolar defect)				0.3016 (0.18-0.41)
10	CCUS vs Composite diagnosis ( Perfusion defect)	66.67% (30-90.32)	100% (97.76-100)	100 (51.01-100)	0.7943 (0.6489-0.9397)
11	CxR vs Composite diagnosis ( Perfusion defect)	50% (18.78-81.24)	87.5% (81.65-91.68)	12.5 (4.344-31)	0.1553 (0.0385-0.2681)
12	CCUS vs CxR (Perfusion defect)				0.10 (0.08-0.207)
13	CCUS vs Composite diagnosis (Metabolic defect)	62.65% (45.25-77.07)	98.59% (95.01-99.69)	90.91 (72.98-97.47)	0.695 (0.55-0.840)
14	CxR vs Composite diagnosis (Metabolic defect)	15.63% (6.864-31.75)	100 (97.37-100)	100 (96.55-100)	0.2321 (0.1369-0.3273)
15	CCUS vs CxR (Metabolic defect)				0.28 (0.15-0.37)

*PPV- positive predictive value

The Cohn’s correlation coefficient between CCUS plus ABG based diagnosis with composite diagnosis was above 0.7 (Table 4). Apart from pneumonia (0.59) and cardiogenic pulmonary edema(0.64) the agreement between two algorithms was very poor (Table 4).

The ability to identify true negatives i.e. specificity was better with CCUS based algorithm as compared to Chest x ray based algorithm in cases of pneumonia 86.49% vs 83.78%, Cardiogenic pulmonary edema 93.28% vs 84.03% (Table 4). The CCUS based algorithm was 100% specific to rule out Pulmonary embolism as compared to 87% for CXR based algorithm. Though the sensitivity of CXR based algorithm was 22.22% sensitive i.e. True positives for detection of ventilation with alveolar defect but specificity was 99.36% (97.36% CCUS). This means that Chest X ray is helpful in ruling out diagnosis of ventilation defect with alveolar defect. Similarly in cases of Metabolic defect , the Chest X ray has specificity of 100%, though sensitivity was 15.3%. An abnormal Chest- X-ray rules out the diagnosis of metabolic defect.

## Discussion

In our study CCUS (which includes Lung ultrasound, Transthoracic echo, IVC assessment and DVT scan) along with inputs from ABG was used to frame an algorithm based diagnosis of dyspneic patients. Round the clock availability of bedside ultrasound and blood gas analyzer helped us in achieving the same without waiting for CXR to make a preliminary diagnosis. The disadvantage of only using LUS has been highlighted by previous authors in which use of Triple scan, i.e., Lung along with IVC and cardiac, has been found to be more informative as compared to LUS alone [9, 10, 11]. In our study we added DVT scan to the Triple scan to aid us further in diagnosis of particularly the perfusion defect as the cause of dyspnoea. We combined CCUS with ABG to make a rapid diagnosis in dyspnoeic patients and also checked efficacy of the same in relation to composite final diagnosis.

The presence of pathological B-lines on LUS points towards two differential’s, i.e pneumonia or pulmonary oedema. The presence of focal B-lines, dynamic bronchogram/ bilateral B-lines with normal Echo points towards diagnosis of pneumonia. On other hand presence of bilateral B- lines with abnormal cardiac contractility points towards diagnosis of pulmonary oedema. We had a group of tachypnoeic patients who had A‐lines ,the differential diagnosis included pulmonary embolism, acute exacerbation of COPD and metabolic disorders. On basis of the CCUS it’s difficult to differentiate as patients with pulmonary embolism and acute exacerbation of COPD could have RA/RV dilatation though mechanisms are different. In case of Pulmonary embolism it’s due to outflow tract obstruction , on other hand in acute exacerbation of COPD its basically due to hypercapnia which leads to increased pulmonary vasoconstriction and leading to RA/RV dilatation. CTPA is the investigation of choice to differentiate these two pathologies but it takes time and is cumbersome. So presence of hypoxemia with hypercarbia point towards diagnosis of acute exacerbation of COPD whereas presence of hypoxemia with hypocarbia points towards pulmonary embolism. Some patients who were nonhypoxic on ABG and have A profile were classified as metabolic causes of dyspnoea. These were patients with sepsis (with site other than lungs), chronic kidney disease, chronic liver disease, and diabetic ketoacidosis. The cause of dyspnoea in such cases is metabolic acidosis which led to increased respiratory drive and thus dyspnoea. In Blue protocol too, there is no mention of metabolic cause of dyspnoea hence ABG was added in the algorithm to differentiate Pulmonary embolism, Acute exacerbation of COPD and Metabolic disorders [7].

Our results of overall diagnostic accuracy of LUS for pneumonia are consistent with recent systematic reviews. In meta-analysis by Staub et al which included 14 studies reported an overall sensitivity of 0.82 (95% CI 0.74–0.88) and specificity of 0.94 (95% CI 0.85–0.98) [4]. Similarly study by Llamas-Alvares et all which included 16 studies in their meta-analysis reported an overall sensitivity of 0.80-0.90 and specificity of 0.70-0.90 [12]. Long L et al in their meta-analysis used lung ultrasound for the diagnosis of pneumonia, reported overall pooled sensitivity and specificity of ultrasound of 0.88 (95% CI: 0.86–0.90) and 0.86 (95% CI: 0.83–0.88) respectively which is similar to our results [13]. We also evaluated agreement between CCUS and CXR based diagnosis of pneumonia and Cohens kappa was found to be 0.59 which indicated that two algorithms had moderate agreement. Haggag YI et al in their study of “Effectiveness of Lung Ultrasound in Comparison with Chest X-Ray in Diagnosis of Lung Consolidation” also calculated the agreement and got a Cohens kappa value of 0.567 (95% CI, 0.422 to 0.712) which was similar to the current study [14]. In critically ill Covid-19 patients admitted with pneumonia lung ultrasound was found useful to predict progression or regression of the disease [15].

Cardiogenic pulmonary oedema is a common cause of ICU admission , Martindale et all assessed the accuracy of LUS for diagnosing heart failure and reported an overall sensitivity of 0.82-0.87 and specificity of 0.910.94, the authors included 8 studies in their metanalysis [16]. Similarly Maw AM et al assessed the diagnostic accuracy of Point-of -Care Lung Ultrasonography and Chest radiography in 1827 patients with Acute Decompensated Heart Failure [17]. The pooled estimates for LUS in diagnosing cardiogenic pulmonary oedema were 0.88 (95% Cl, 0.75-0.95) for sensitivity and 0.90 (95% Cl, 0.88-0.92) for specificity. Pooled estimates for CXR were 0.73 (95% CI, 0.70-0.76) for sensitivity and 0.90 (95% CI, 0.75-0.97) for specificity. In our study out of 55 patients labelled as cardiogenic pulmonary oedema by composite diagnosis 52 patients were correctly identified by CCUS giving a sensitivity of 94.55% and specificity of 93.28%. Using the CXR based algorithm had a sensitivity of 90.91% and specificity of 84.03%. The relative improvement in the sensitivity and specificity of diagnosing the defect could be attributed to the algorithmic based approach including ABG analysis along with CCUS which gave us a more comprehensive picture. We also evaluated agreement between CCUS and CXR based diagnosis of CPE, Cohens kappa was found to be 0.6438 which indicated that two modalities had substantial agreement.

Acute exacerbation of chronic obstructive pulmonary disease is a common emergency and the most common precipitating factor is usually respiratory tract infection [18]. In meta- analysis by Staub etal which included 4 studies, the presence of A-profile without PLAPS (posterior- lateral alveolar pleural syndrome) on LUS had sensitivity of 0.78 (95% CI 0.67–0.86) and specificity of 0.94 (95% CI 0.89– 0.97) for Acute exacerbations of COPD [ 19-21]. In our study the CCUS and ABG based algorithm had a sensitivity of 83.33% with 95% CI (60.78, 94.16) and specificity of 97.44% with 95% CI (93.59,99.00), also having a high negative predictive value of 98.06% with 95% CI (94.46, 99.34) implying the fact that CCUS and ABG based algorithm can safely rule out diagnosis of Acute Exacerbation of COPD. The sensitivity and specificity both are higher as compared to LUS alone, as presence of A Profile without PLAPS could be feature common to pulmonary embolism and metabolic disorders. CXR based algorithm only identified correctly 4 out of 18 patients labelled as COPD with pneumonia by composite diagnosis thus having a sensitivity of only 22.22%. We also evaluated agreement between CCUS and CXR based diagnosis of COPD with pneumonia and Cohens kappa was found to be 0.3016 (CI 0.18-0.41) which indicates that two modalities didn’t have a good agreement. Thus we can conclude that CCUS and ABG based algorithm is an excellent modality for diagnosis of Acute Exacerbation of COPD as compared to Chest-X- ray based diagnosis.

The gold standard test for diagnosis of pulmonary embolism is CT pulmonary angiography, as it’s not readily available and if available it’s difficult particularly for a hemodynamically unstable patient to be shifted for CT angiography [22] . Comert SS et al in their study, Role of thoracic ultrasonography (TUS) in the diagnosis of pulmonary embolism, reported that out of 50 patients with suspected PE, PE was diagnosed in 30 patients [22]. It was shown that TUS was true positive in 27 patients and false positive in eight and true negative in 12 and false negative in three. Sensitivity, specificity, positive predictive value, negative predictive value, and diagnostic accuracy of TUS in diagnosis of PE for clinically suspected patients were 90%, 60%, 77.1%, 80%, and 78%, respectively [23]. Zotmann et al combined LUS with Well’s score in Covid 19 patients to detect pulmonary embolism and found that Well’s score of more than 2 had a good predictive value [24]. In our study out of 6 patients labelled as PE by composite diagnosis 4 (66.67%) patients were correctly identified by CCUS plus ABG based algorithm with sensitivity of 66.67% and specificity of 100%. Though sensitivity of our algorithm was lower as but specificity was spot on, we are unlikely to miss true negatives. Probably in this addition of ABG would have helped as all those patients who had higher PaCO_2_ wouldn’t have Pulmonary embolism. We have also evaluated agreement between CCUS and CXR based diagnosis of PE and Cohens kappa was found to be 0.10 (CI 0.0080.207) which indicates that two modalities have poor agreement.

Among Metabolic disorders, we have included other common aetiologies of dyspnoea requiring ICU admission like Sepsis, Toxidromes, Diabetic ketoacidosis and Neuromuscular disorders. To the best of our knowledge there have been no studies on diagnosing these pathologies using ultrasound or CXR. Out of 32 (18.39%) patients labelled as metabolic defect by composite diagnosis 20 patients were correctly picked by CCUS plus ABG based algorithm with sensitivity of 62.5% and specificity of 98.59%. Though sensitivity was lower but had a high specificity, hence presence of abnormal LUS rules out the diagnosis of metabolic disorder. On the other hand by using the CXR based algorithm only 5 out of total 32 patients under metabolic category were correctly identified. We have also evaluated agreement between CCUS and CXR based diagnosis of PE, Cohens kappa was found to be 0.26 (CI 0.15-0.37) which indicated that two modalities did not had good agreement.

Total 89 patients needed intubation out of which 33 patients belonged to the alveolar defect (pneumonia) category. Of the 89 patients, 43 were weaned off from ventilator and discharged from ICU while 46 succumbed. Of the 43 patients who were successfully extubated, 21 belonged to the alveolar defect (pneumonia) category. Thus 21 (63%) out of the 33 patients belonging to alveolar defect (pneumonia) category were extubated which was highest among all the five pathophysiologic categories. 44 of 59 ( 74.57%) patients had on admission SOFA score of 10 or more, 17 out of these 44 patients (SOFA>10) had alveolar defect(cardiac) and all the 17 patients died during their ICU stay thus underlining the importance of early diagnosis and initiation of treatment in patients with alveolar defect (cardiac) and having a higher SOFA score. Apart from diagnosis this CCUS plus ABG based algorithm also pre-empts that what mode of ventilation will be needed for these patients. As patients with alveolar pathology (pneumonia) will most of the time land up getting invasive mechanical ventilation, patients with alveolar (cardiac) pathology will be settled on NIV, patients with Acute exacerbation of COPD will be managed on NIV while those with pulmonary embolism and metabolic defects mode of oxygen therapy can vary as per the disease severity.

Few of our patients had combined pathophysiology i.e. Alveolar (cardiac) along with Alveolar (pneumonia) but were eventually counted in pneumonia group. We didn’t have any patient of pneumothorax, pulmonary contusion during study period which could also present to hospital with complaints of dyspnoea. We didn’t create a pathophysiological heading of atelectasis in our study, in recent study by Haaksma et al authors demonstrated that LUS can easily differentiate atelectasis from pneumonia [25]. This study was limited to around 200 patients and was a single centre study, large centre multicentric trials would further validate our results.

This is a first of it’s kind study, where authors have attempted combining two point of care tests i.e. bed side ultrasound and ABG for diagnosing aetiology of dyspnoea and creating an algorithmic approach for timely diagnosis and intervention. For acute exacerbation of COPD, CCUS plus ABG based algorithm had a specificity of near 97% which is encouraging. Though number of patients with pulmonary embolism was less, the specificity for diagnosis of pulmonary embolism was cent percent. We also on the basis of combination of CCUS and ABG attempted to diagnose metabolic disorders which is not possible with LUS alone.

## Conclusion

CCUS plus ABG algorithm is highly sensitive and it’s agreement with composite diagnosis is far superior. The addition of ABG component as compared to LUS alone has higher sensitivity and specificity for diagnosing acute exacerbation of COPD , higher specificity for pulmonary embolism . The addition of ABG component helps us to identify metabolic disorders which are not picked up by LUS alone.
